# Evaluation of functional rehabilitation physiotherapy protocol in the postoperative patients with anterior cruciate ligament reconstruction through clinical prognosis: an observational prospective study

**DOI:** 10.1186/s13104-016-2234-9

**Published:** 2016-09-23

**Authors:** Tabata Cristina do Carmo Almeida, Luiz Vinicius de Alcantara Sousa, Diego Monteiro de Melo Lucena, Francisco Winter dos Santos Figueiredo, Vitor Engrácia Valenti, Laércio da Silva Paiva, Luiz Carlos de Abreu, Fernando Adami

**Affiliations:** 1 Faculdade de Medicina do ABC, Laboratório de Epidemiologia e Análise de dados, Departamento de Saúde da Coletividade, Av. Príncipe de Gales, 821, Príncipe de Gales, CEP: 09060-650 Santo André, SP Brazil; 2 Faculdade de Medicina do ABC, Laboratório de Delineamento de Estudos e Escrita Científica, Departamento de Saúde da Coletividade, Av. Príncipe de Gales, 821, Príncipe de Gales, CEP: 09060-650 Santo André, SP Brazil; 3 Escola de Filosofia e Ciências., Universidade Estadual Paulista Júlio de Mesquita Filho, Rua Quirino de Andrade, 215, CEP: 01049-010 Marília, SP Brazil

**Keywords:** Rehabilitation, Ligaments, Anterior cruciate ligament

## Abstract

**Background:**

The aim of the study was to evaluate the evolution of patients subject to physical treatment based on guidelines of functional rehabilitation after surgery anterior cruciate ligament reconstruction.

**Methods:**

This is a prospective study of 177 patients with anterior cruciate ligament injury, who underwent surgery and physical therapy guideline conducted in an orthopedic clinic in São Paulo, southeastern Brazil. The clinical evolution of patients was made according to Lysholm and IKDC questionnaire on the 1st day after surgery with 30, 90 and 180 days of treatment.

**Results:**

There was statistically significant increase in the gross values of Lysholm and IKDC questionnaires during the treatment (p < 0.001), which indicates progressive gain of function. According to the scores obtained from the IKDC, it can be observed that in stage 1 the average progress was 53.5 %, falling to 50 % in stage 2, and 26.1 % in stage 3. As to Lysholm score, it started with 87.7 %, falling to 62.6 % in the second stage and 7 % in the third stage, both statistically significant (p < 0.001). The rehabilitation-oriented functional objectives priority is to quickly get the exercises to gain breadth, strength and proprioception, optimizing and improving the integration of the athlete back to sport.

**Conclusion:**

Synthesizing the gradual gain of function and according to clinical outcomes assessed by IKDC and Lysholm, the functional guideline presented may be considered an alternative for rehabilitation of patients in postoperative anterior cruciate ligament.

## Background

The incidence of anterior cruciate ligament injury among the American population associated to sports practice is 3 per 10,000 inhabitants, and approximately one hundred thousand surgeries for the reconstruction of such ligament are performed in the United States on a yearly basis [1–3].

Normal operation of the lower limbs is essential both for the activities of daily life and for sports. The interest in the knee joint, particularly the anterior cruciate ligament (ACL) reconstruction has increased more and more, since the disease and its treatment is a challenge for many health care professionals connected to this tematic [1–5].

In relation to the cruciate ligament injury most authors agree that it results in biomechanical abnormalities, abnormal function and knee kinematics. On the other hand, several studies have shown conflicting results in physical therapy and rehabilitation programs. The work developed is based on professional experiences or focuses on specific aspects of rehabilitation. For this reason, we did not find statistical or a follow-up long-term data to prove the conclusions reached with physical therapy rehabilitation programs realized [1–5].

Physical therapy plays an important role in the recovery of these patients, as several studies have been developed to support clinical guidelines that must be followed and thus enable effective and updated treatment, which can solve the deficiencies, normalize the static stability and knee dynamic and rehabilitate in the shortest time possible, but in a highly secure manner. The first treatments, entitled conventional, emphasized the protection of the graft restricting movement and increasing the turnaround time activities. The increased incidence of joint stiffness in these postoperative led to further studies and changes in protocols [3–6].

Later studies developed the accelerated protocols showing that the knee mobilization and its early strength did not compromise the graft healing, knee stability and even decreased the patients recovery time [7, 8]. Currently other studies have been conducted emphasizing that, in addition to earliness of movements, there is a need for full functional recovery and is directed to the patients individual needs [8–11].

Although several lines of research related to physical therapy, it is known that the main difference between treatment protocols has been the temporal duration. On the other hand, we did not find in the literature consensus on the effectiveness of treatments or better type of protocol to be followed [11, 12]. The aim of the study was to evaluate the application of a functional protocol physical therapy rehabilitation in patients with ACL reconstruction based on functional clinical prognosis.

## Methods

An observational prospective study conducted in a specialized orthopedic clinic from January 2006 through December 2011. The clinic is located in São Paulo State at Santo André city in Brazil. The study was approved by the independent Committee of ethics of the Health Department of Santo André (022/2011).

The population of the study began with 250 patients in physiotherapy treatment for postoperative anterior cruciate ligament reconstruction. Study participants were recruited from the specialized clinic at Santo André city through the inclusion criteria: (i) Anterior cruciate ligament reconstruction surgery in only one knee (ii) patients were on treatment for the first anterior cruciate ligament surgery (iii) patients completed all evaluation questionnaires (iv) patients attend to physical therapy at least four times a week during the studies. Information as social, demographic and clinically related characteristics (gender, age, type of graft, injured knee, sports practiced) were collected through medical records at the clinic.

All participants signed an informed consent. All the information was compiled to create a database of patient characteristics and treated with ethical research procedures to assure privacy during data collection and throughout treatment. The functional rehabilitation guideline performed on patients, was developed by the authors protocol.

The protocol used in this study is composed of three steps of an exercise program with goals to be achieved at each step before moving forward to the next step of the protocol.

First step: to reduce pain and inflammation, restore range of motion of the knee, and optimize muscle control, especially in March.

Second step: to intensify the muscular and sensory-motor rehabilitation.

Third step: to strengthen the surrounding muscles and sensory-motor rehabilitation through more intense activities.

When evaluating the data, was required all patients to complete all evaluation questionnaires and the physical therapy for at least four times a week during the study. The final analysis of this article was based on 177 patients as they attended to all the inclusion criteria.

To evaluate the patients during the treatment, it was used two questionnaires: International Knee Documentation Committee (IKDC) and Tegner Lysholm Scoring Scale.

The IKDC is a questionnaire comprised of ten questions, divided into three domains. The first domain assesses symptoms such as pain, stiffness, swelling, locking and giving way feeling. The second domain assesses sport and daily activities. The third domain evaluates the function of the knee before and during the injury. The IKDC is marked via the calculation of the difference between the gross value and the lowest score possible, and then by dividing such difference by the range of possible scores multiplied by 100. The items are added up to produce a single index with higher values indicating higher function levels and minor knee symptoms [13, 14].

The Tegner Lysholm Knee Scoring Scale assesses signs and symptoms. It is comprised of 8 multiple-choice questions that assess limp, pain, locking of the knee, stair climbing, support, instability, swelling, and squatting [13, 15]. The questionnaires were applied by a direct interview with the patients at four time points during the physical therapy treatment: a day post-surgery, 30 days of treatment, 90 days of treatment, and 180 days of treatment. Then, questionnaires were evaluated into IKDC and Lysholm scores as: *IKDC* group 1 (score <41), group 2 (≥41 and <81), group 3 (≥81 and <100), group 4 (=100) [14, 16, 17], *Lysholm* poor (score <65), fair (≥65 e <84), good (≥84 e <91), excellent (≥91) [15].

All the qualitative variables were presented by relative and absolute frequency. Friedman’s test was used to compare the distribution of the scores obtained from the IKDC and Lysholm over the 180 days. The statistical analysis software used was Stata 11.0.

## Results

Table 1 shows that among the 177 participants, there was a higher prevalence of lesions in men (81.4 %), right knee was operated on (65.5 %) of the cases, football (soccer) was being practiced on (65 %) of the participants at the lesion moment and (92.1 %) of the study surgeries had semitendinosus tendon used as graft surgery.

**Table 1:** Description of patients with anterior cruciate ligament reconstruction that underwent treatment functional physical therapy guideline. Brazil, 2006–2011

Variables	n (%)
Sex	
Male	144 (81.4)
Female	33 (18.6)
Knee operated	
Right	116 (65.5)
Left	61 (34.5)
Sport being practiced when injury occured	
Soccer	115 (65.0)
Volleyball	16 (9.0)
Tennis	10 (5.7)
None	23 (13.0)
Others	13 (7.3)
Type of surgical	
Semitendinosus	163 (92.1)
Patellar tendon	14 (7.9)
Total	177 (100)

Table 2 shows that according to the found results after the day after the surgeries, more than (93.8 %) of the patients presented poor scores according to Lysholm and (95.5 %) of the patients presented poor scores (group 1) according to IKDC.

**Table 2:** Distribution of patients undergoing functional guideline after surgical reconstruction of the anterior cruciate ligament, according to the classification obtained from Lysholm and IKDC questionnaires

Day	Lysholm			
	Poor n (%)	Fair n (%)	Good n (%)	Excellent n (%)
Day 1	166 (93.8)	11 (6.2)	0 (0.0)	0 (0.0)
Day 30	140 (79.1)	34 (19.2)	3 (1.7)	0 (0.0)
Day 90	0 (0.0)	17 (9.6)	45 (25.4)	115 (65.0)
Day 180	0 (0.0)	0 (0.0)	9 (5.1)	168 (94.9)

Day	IKDC			
	Group 1 (<41) n (%)	Group 2 (≥41 and <81) n (%)	Group 3 (≥81 and <100) n (%)	Group 4 (=100) n (%)
Day 1	169 (95.5)	8 (4.5)	0 (0.0)	0 (0.0)
Day 30	59 (33.3)	118 (66.7)	0 (0.0)	0 (0.0)
Day 90	0 (0.0)	158 (89.3)	19 (10.7)	0 (0.0)
Day 180	0 (0.0)	3 (1.7)	130 (73.5)	44 (24.9)

The first classifications indicates the presence of symptoms (measured especially by the Lysholm score) and function impairment (measured mainly by the IKDC). A considerable increase on test scores was observed on the 30th, 90th and 180th days of treatment among participants of the study, which indicates a positive evolution on decreasing the presence of symptoms and increasing the gain of function. It was also observed that only a small percentage Lysholm (5.1 %) and IKDC (1.7 %) of the patients had not reach the highest score at the tests in the end of the 180 days of treatment, in other hand (24.9 %) reached the maximum score of the IKDC form, which implies in full function recovery.

**Fig. 1:**
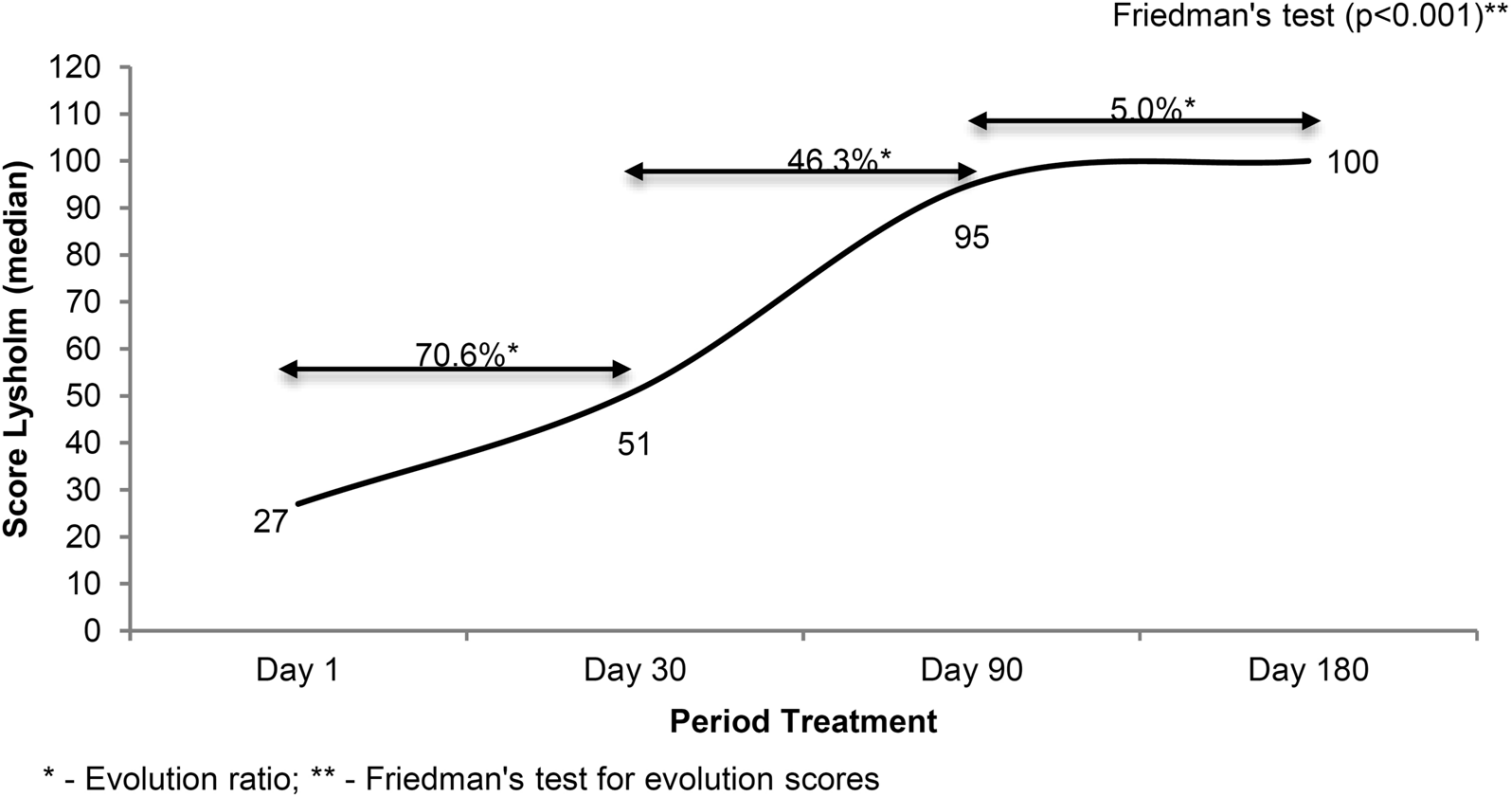
Evolution of Lysholm scores of patients undergoing physiotherapy functional post-surgery protocol reconstruction of the anterior cruciate ligament, Santo André, Brazil

**Fig. 2:**
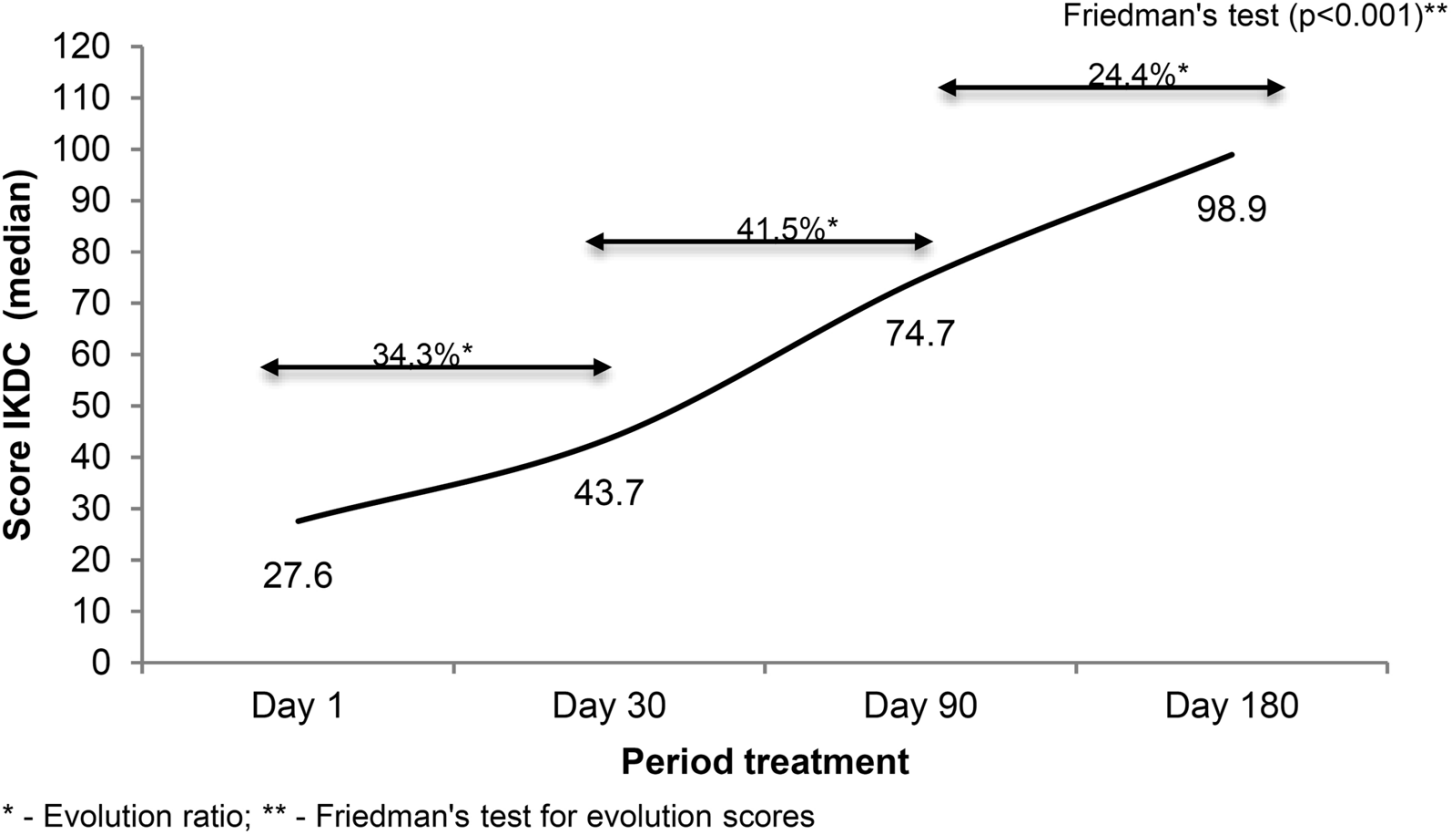
Evolution of IKDC scores of patients undergoing physiotherapy functional post-surgery protocol reconstruction of the anterior cruciate ligament, Santo André, Brazil

Figures 1 and 2 shows that there was a significant statistically increase on Lysholm and IKDC questionnaires gross values during the treatment (p < 0.001), which indicates progressive gain of function.

Figure 1 shows the progress of patients according to the Lysholm scale. The median between 1st, 30th, 90th and 180th days (median 27, 51, 95 and 100 respectively), and Fig. 2 shows the scale according to the IKDC, 27.6 median for the 1st day, 43.7 for the 30th day, the 90th day to 74.7 and 98.9 for the 180th day. The evolution of the knee functionality was statistically significant at both Lysholm scale (p ≤ 0.001) and IKDC (p ≤ 0.001) from the first to the last day established by the guideline.

The Lysholm scale shows a stronger growth on the first 30 days (70.6 %), while the IKDC scale stronger growth is showed from 30th to 90th days (41.5 %).

Although both smaller scales shows evolution from 90th to 180th, the observed evolution on Lysholm scale was minor than that the evolution found in IKDC scale (5 and 24.4 %, respectively).

## Discussion

The principal findings of this study were:

1. The majority of patients undergoing this guideline treatment protocol evolved from bad to excellent in the Lysholm scale (94.9 %)
2. From 177 patients studied (95.5 %) started with a score <41 and at the end of the study (98.45 %) reached the top 81 in the IKDC score scale
3. A considerable increase on test scores was observed for this guideline, which indicates a positive evolution on decreasing the presence of symptoms in the first 30 days showed by Lysholm scale (70.6 %) and increasing the gain of function among 30–90 days in the IKDC scale (41.6 %).

According to the found results there was a significant statistically increase on gross values for Lysholm and IKDC, which suggests a progressive gain of functionality.

Considering that the treatment guide is a new conjunction of scientific assumptions about ACL rehabilitation, we have not found studies with similar series to allow an evolution comparison of the values found on IKDC and Lysholm about this prognostic.

The used questionnaires to evaluate the prognosis in this patients despite evaluating symptoms and function together, do not compromise the final scores, as any motor function depends on an intact sensory motor system to be performed, which includes muscle strength, mobility and neuromuscular control [18, 23, 24]. Pain is a limiting factor to perform any physical activity, however when a knee is injured several receptors and neural feedback pathways are injured as well, therefore absence of pain does not indicate proper motor skills, which is indicated with low grades in the questionnaires.

Most of the patients presented impairment of function and symptoms, according to the scores of IKDC and Lysholm. These results are consistent with those reported in the literature and are justified by the fact that both instability and pain are very typical symptoms in this type of ligament injury, as described Peccin et al. [15], Bonfim [19] and Mir et al. [20]. reported after the anterior cruciate ligament reconstruction there is a loss of knee function due to an altered proprioceptive response of the new ligament.

It is believed that gradual adaptation in function gain is the main way to avoid complications throughout the rehabilitation process; constant evaluations will direct treatment for current deficits and can improve the final results of the treatment.

At the end of the 180 days of treatment, more than 90 % of patients presented high scores, which is clinically equivalent to a good functional recovery [18]. Authors such as Collins et al. [13], Hambly and Griva [16] and Metsavaht [14], believes that high scores allows the patients return to sports practice, as these patients do not have functional complaints regarding restriction or symptoms.

Scores with a better functional recovery were observed after 180 days of treatment, which is consistent with what literature reports of approximately 6 months for ACL rehabilitation. This also evidences that regardless of the gender, kind of sport, and the fact of practicing a physical activity or not, the rehabilitation stages remained unchanged [21, 26].

In the first stage, the progress rate according to the IKDC was 53.5 and 87.7 for the Lysholm score. In the third stage it was 26.1 and 7, respectively. Studies presenting the same results were not found in the literature, but it is believed that this has probably happened because in the beginning of treatment, the symptoms and limitations are evident and as treatment goes by the focus turns to be the optimization of static and dynamic stability, which have slower physiological responses [23–25].

The presented guideline corroborates the work of Yabroudi and Irrgang [22], Gerber et al. [9] and Myer et al. [8] who defends the idea that rehabilitation programs should reflect the advancement of surgical and clinical procedures. However the established guidelines for graft and career progression protection must be respected and followed

With the scores obtained with the IKDC and Lysholm in this study, we can see that the rehabilitation guided by functional objectives can improve the patient integration, who is can return to sport, as strength, functional stability of the knee, activity symmetry, resistance, symptoms, agility, and sporting gestures are assessed through these questionnaires and the guideline itself. Additionally, the functional approach has the advantage of helping patients to gradually adapt to their needs, as each successive stage is based on the previous ones, besides it measures and identifies the deficits that can be used to justify the restrictions or limitations at any time during the treatment.

## Conclusion

Given the values obtained with the IKDC and Lysholm in treating patients with reconstruction of the anterior cruciate ligament (ACL), there is a satisfactory functional outcome of these patients suggesting that this protocol may be a physical therapy treatment alternative to be used.
